# Generalism and specialism: Reasserting the value of specialist knowledge in an era of multimorbidity

**DOI:** 10.1016/j.fhj.2026.100548

**Published:** 2026-06-25

**Authors:** Waqas Akhtar, Luigi Camporota

**Affiliations:** Guy’s & St Thomas’ NHS Foundation Trust, Westminster Bridge Road, London SE1 7EH, United Kingdom

**Keywords:** Generalism, Specialism, Medical training

## Abstract

Emphasis has increasingly been placed on expanding medical generalism in response to an ageing population and the rising prevalence of multimorbidity. While the ability to manage complex, undifferentiated illness across multiple conditions is undeniably important, modern healthcare is simultaneously becoming more technologically advanced and clinically specialised. Breakthroughs in diagnostics, therapeutics and procedural medicine demand deep, focused expertise to deliver safe and effective care, particularly for high-risk patients. Rather than viewing generalism and specialism as competing models, healthcare systems must recognise them as complementary. Generalists are essential for holistic assessment, coordination and continuity, while specialists provide advanced interventions and nuanced disease-specific management. In an era defined by both multimorbidity and rapid medical innovation, the central challenge is not to prioritise one approach over the other, but to strike an appropriate balance which ensures that patients receive the right expertise at the right time.

There has been great interest in valuing and expanding medical generalism,^1^ building on the 2023 chief medical officer’s report.^2^ The case for strengthening medical generalism is at first glance compelling, particularly in the context of an ageing population with increasing complexity of multimorbidity, fragmentation of care across specialties and primary care, and need for continuity and whole-person care. However, we wonder whether this framing risks distracting from the underlying problem facing the NHS and proposing a solution that may dilute expertise, reduce quality, and fail to meet the growing complexity of modern healthcare.

A central ambiguity in the debate is what is actually meant by ʻgeneralism’. Much of what is described as generalism is already embedded within undergraduate medical education and early postgraduate training. Medical school, foundation programmes and core rotations are explicitly designed to produce doctors with broad generalist knowledge: how to assess undifferentiated illness, manage uncertainty, prioritise risk and coordinate care. In our view, this definition of generalism is a foundational competency, not a neglected or newly emerging domain.

True generalists are indispensable. Managing the undifferentiated patient, recognising serious illness among non-specific presentations, and knowing when and how to involve specialist teams are core skills of doctors such as general practitioners, emergency physicians or geriatricians. No amount of additional ʻgeneralist’ training enables a cardiologist, neurologist or gastroenterologist to replicate the work of an emergency department doctor, just as no degree of subspecialty exposure allows a generalist to replace specialist expertise. These roles are distinct, complementary and equally necessary.

The problem arises when generalism is presented not as a clearly defined professional strength with a need for more generalists, but as a training solution for specialists. Increasing time spent delivering general medical service has already reduced opportunities for specialist trainees to acquire the skills required for modern practice, as seen clearly in cardiology^3^ and other specialties. This is not theoretical: procedural volume, clinic exposure and subspecialist development have already been eroded. Many resident doctors now require post-certificate of completion of training (CCT) fellowships to be even eligible for consultant posts.^4^ The UK is already an outlier internationally in the extent to which specialists are expected to deliver general medicine during training.^5^ Expanding this further risks undermining specialist capability without meaningfully strengthening generalist care.

Medicine has become increasingly specialised for good reasons. Advances in diagnostics, interventions and therapeutics demand clinicians with deep expertise in increasingly complex and narrow fields. Multimorbidity does not simply mean ʻmany conditions’; it often involves interacting, high-risk disease states requiring deep pathophysiological understanding and nuanced decision-making. In such cases, harm can result not from excessive specialisation, but, on the contrary, from delayed or insufficient specialist input. This is not to deny that specialists themselves require broad clinical judgement. Caring for patients with multimorbidity demands the ability to manage diagnostic uncertainty, weigh competing priorities and reason across organ systems. These generalist competencies are essential to good specialist practice, but they are cultivated within specialist training rather than serving as a substitute for it.

This is particularly relevant given how the care of older patients has changed. Advanced age is rightly no longer a barrier to intervention. Patients in their 80s and 90s now routinely undergo complex cardiological procedures, advanced oncological therapies, renal replacement strategies and invasive diagnostics. High-technology medicine is expanding into older populations, not retreating from them. Delivering this safely requires specialist-led assessment and management.

Expecting individual clinicians to maintain up-to-date expertise across multiple rapidly advancing fields risks superficial decision-making and overly conservative care that neglects evidence-based, personalised treatment. The appeal of ʻexpert generalists’ often reflects frustration with poor patient flow, delayed discharge and lack of continuity. Yet these reflect organisational and structural failures, such as bed shortages, weak community provision and fragmented information systems, rather than inherent flaws of specialism.

There is also a strategic risk. The NHS’s ability to improve outcomes, adopt innovation and contribute to global medical research depends on deep specialist expertise as much as generalist expertise. A workforce skewed too heavily towards generalism risks reduced specialist research leadership, slower adoption of new therapies and diminished international standing.

The solution is not to blur roles, but to respect them. Generalists should be enabled to build on their strengths, and specialists theirs. While the NHS may indeed need more generalists, it does not need to expand generalism training for specialists. The system needs both, functioning in balance. This is alongside better protection of training time, improved interfaces between specialties and primary care for shared care pathways.

The future NHS will succeed not by asking all doctors to know a little about everything, but by ensuring that patients consistently receive the right expertise, at the right time, from the right doctor.

## CRediT authorship contribution statement

**Luigi Camporota:** Writing – review & editing, Conceptualization. **Waqas Akhtar:** Writing – original draft, Conceptualization.

## Funding

This article did not receive any specific grant from funding agencies in the public, commercial, or not-for-profit sectors.

## Declaration of Competing Interest

The authors declare that they have no known competing financial interests or personal relationships that could have appeared to influence the work reported in this paper.
